# Engineering a fidelity-variant live-attenuated vaccine for chikungunya virus

**DOI:** 10.1038/s41541-020-00241-z

**Published:** 2020-10-14

**Authors:** Christopher M. Weiss, Hongwei Liu, Kasen K. Riemersma, Erin E. Ball, Lark L. Coffey

**Affiliations:** 1 Department of Pathology Microbiology and Immunology, University of California, Davis, CA USA; 2 Present Address: University of Wisconsin, Madison, WI USA

**Keywords:** Viral infection, Live attenuated vaccines, Alphaviruses

## Abstract

Chikungunya virus (CHIKV), which causes a febrile illness characterized by severe and prolonged polyarthralgia/polyarthritis, is responsible for a global disease burden of millions of cases each year with autochthonous transmission in over 100 countries and territories worldwide. There is currently no approved treatment or vaccine for CHIKV. One live-attenuated vaccine (LAV) developed by the United States Army progressed to Phase II human clinical trials but was withdrawn when 8% of volunteers developed joint pain associated with vaccination. Attenuation of the Army’s CHIKV LAV strain 181 clone 25 (CHIKV-181/25) relies on two mutations in the envelope 2 (E2) glycoprotein responsible for cell binding and entry, making it particularly prone to reversion, a common concern for replication-competent vaccines. High error rates associated with RNA virus replication have posed a challenge for LAV development where stable incorporation of attenuating elements is necessary for establishing safety in pre-clinical models. Herein, we incorporate two replicase mutations into CHIKV-181/25 which modulate CHIKV replication fidelity combined with additional attenuating features that cannot be eliminated by point mutation. The mutations were stably incorporated in the LAV and did not increase virulence in mice. Two fidelity-variant CHIKV LAVs generated neutralizing antibodies and were protective from CHIKV disease in adult mice. Unexpectedly, our fidelity-variant candidates were more mutable than CHIKV-181/25 and exhibited restricted replication in mice and *Aedes* mosquitoes, a possible consequence of hypermutation. Our data demonstrate safety and efficacy but highlight a further need to evaluate fidelity-altering phenotypes before use as a LAV given the potential for virulent reversion.

## Introduction

Chikungunya virus (CHIKV) is a mosquito-borne alphavirus, which causes an acute febrile illness characterized by polyarthralgia, polyarthritis, maculopapular rash, and myalgia^1,2^. Symptoms typically resolve within 1–2 weeks, but about 1–4% of cases result in chronic flaring joint pain that can persist for months or years after infection^3–7^. In rare cases, neuroinvasive disease and other complications have been observed, leading to fatality^8–11^. CHIKV was first identified in East Africa but has since spread across Asia and the Americas with autochthonous transmission implicated in multiple European outbreaks of the disease as well^1,12–15^. Travel-associated cases are imported to the United States annually from endemic regions, and a brief period of autochthonous transmission was observed in Florida in 2014 and Texas in 2015^16^. There are currently no approved vaccines or targeted therapeutics available for the prevention or treatment of chikungunya disease.

The emergence of CHIKV on a global scale has highlighted the urgent need for a licensed vaccine. Current clinical evaluation is underway for CHIKV vaccines including a purified virus-like particle (VLP) lacking replicating genetic material^17^, measles virus-vectored VLP^18^, a replication-competent CHIKV strain with a large deletion in a non-structural gene^19^, and an attenuated, infectious DNA (iDNA)-launched CHIKV with a restricted mutant spectrum^20^. The first CHIKV vaccine to be tested in humans, CHIKV-181/clone 25 (CHIKV-181/25), was generated by serially passaging a human CHIKV isolate from Thailand 18 times in human lung fibroblast cells^21^. Attenuation of CHIKV-181/25 is conferred by two point mutations (T12I and G82R) in the E2 envelope glycoprotein responsible for cell surface receptor recognition^22^. Despite apparent genetic stability in subsequent passages and no safety concerns raised by Phase I clinical trials, CHIKV-181/25 inoculation resulted in transient arthralgia in 8% of healthy volunteers who were administered the vaccine in Phase II clinical trials^21,23^. Characterization of the attenuating mutations in isolation in mice revealed partial reversion to the parental virus sequence, suggesting a potential explanation for the observed adverse events following vaccination^22^.

A risk of virulent reversion is inherent to any live-attenuated vaccine (LAV) candidate and poses a significant barrier to large human trials and regulatory agency approvals. RNA viruses like CHIKV are at heightened risk of reversion due to error-prone replication from RNA-dependent RNA polymerases (RdRPs), which usually lack the proofreading capacities possessed by DNA polymerases. The typical viral RdRP incorporates an erroneous nucleotide during replication at a rate of 1 per 10^3^ to 10^5^ nucleotides^24^, versus 1 in 10^4^–10^6^ nucleotides by non-error-correcting DNA polymerases^25^. Furthermore, mismatch repair mechanisms help drive DNA replication error rates down to just 1 in 10^6^–10^8^ nucleotides^25^. Replication fidelity of RdRPs can be manipulated through serial passage in the presence of nucleoside analogs, artificially selecting for polymerases that more stringently incorporate target nucleotides^26–29^. We previously reported on two such mutations in the CHIKV replication complex, RdRP mutation nsP4^C483Y^, and protease/helicase mutation nsP2^G641D^ ^26,30^. Combined, these replication complex variants reduce the observed CHIKV error rate by as much as 50% when assessed by targeted bacterial cloning-based sequencing of the envelope 1 (E1) surface glycoprotein^26^. Recently, however, our lab employed next-generation sequencing (NGS) approaches to resolve error type and frequency derived by these variants at greater resolution with mixed results^30^. While the antimutator phenotype of CHIKV strain 06–049 nsP2^G641D^/nsP4^C483Y^ (CHIKV-06-049-P2.P4) is maintained in targeted sequencing approaches, a broad survey of nucleotide substitution rates measured by NGS revealed elevated error incorporation in progeny virus populations compared to the wild-type sequence, raising concerns that the sequencing methodology used can influence antimutator assignation.

Fidelity-altering mutations have previously been demonstrated to have a negative impact on viral fitness, with increased replication fidelity associated with RNA virus attenuation^26,28,31^. The attenuating nature of these mutations, coupled with the potential reduction in LAV replication errors, have made fidelity variants a promising approach for rational vaccine design^32–34^. Herein we report on the incorporation of the previously described fidelity-altering mutations nsP2^G641D^/nsP4^C483Y^ in the live-attenuated vaccine, CHIKV-181/25, as a method for modulating replication errors. In addition to the antimutator variant, we generated additional vaccine candidates incorporating other known attenuating elements. These include a picornavirus internal ribosomal entry site (IRES) previously described to attenuate both Venezuelan equine encephalitis virus (VEEV) and CHIKV^35–41^ and a combined deletion of the envelope 3 (E3) C-terminal domain and a homologous rescue mutation in the E1 glycoprotein, previously also described as an attenuator of VEEV^42–44^. These LAV CHIKV vaccine candidates were used to compare murine virulence, protection, immunogenicity, and genetic stability.

## Results

### Fidelity-variant mutations do not compromise the viability of CHIKV LAV

**Fig. 1:**
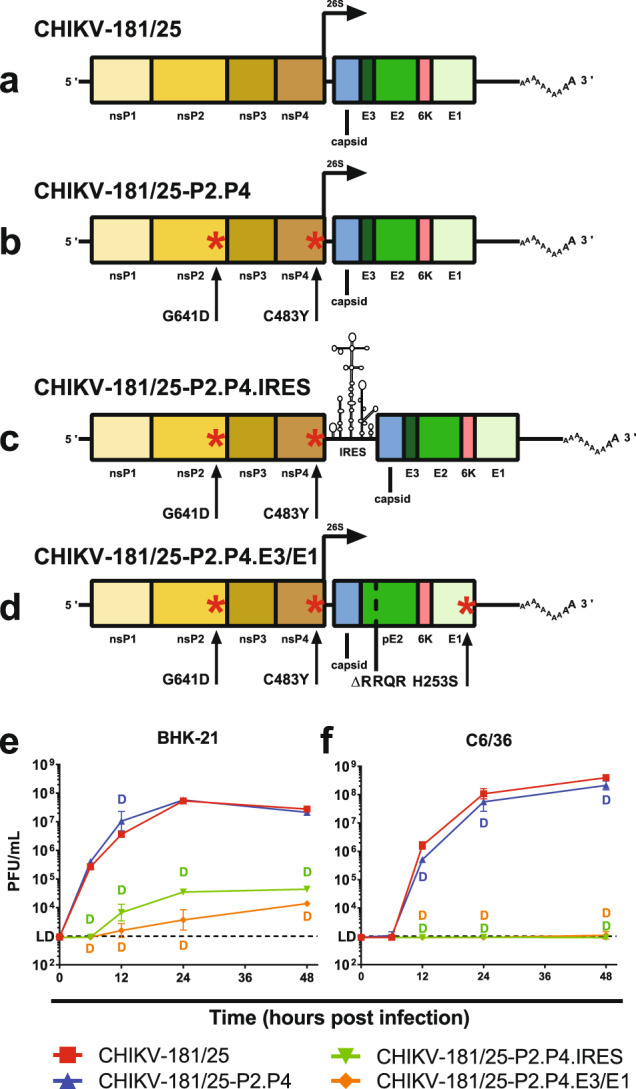
CHIKV live-attenuated vaccine design and replication kinetics. The organization of CHIKV vaccine candidates is shown with point mutations denoted by a red asterisk (**a**–**d**). Live-attenuated vaccine growth is demonstrated on vertebrate baby hamster kidney (**e**) and mosquito *Aedes albopictus* larval (**f**) cells at starting MOI = 1. *P* < 0.0001 (D), 2-way ANOVA of log-transformed values, *n* = 3 per experiment, two combined experiments. Geometric mean and geometric standard deviation are indicated with data points and error bars (**e**, **f**).

Mutations to the CHIKV replicase in both the RdRP (nsP4) and protease/helicase (nsP2) have been previously reported to improve replication fidelity between successive rounds of viral replication with additive effect. To minimize the mutability of CHIKV-181/25, the US Army LAV (Fig. 1a), we incorporated two mutations, nsP2^G641D^ and nsP4^C483Y^, in an infectious clone of CHIKV-181/25 to generate the fidelity-variant CHIKV-181/25-P2.P4 (Fig. 1b).

As added vaccine attenuation measures, two additional CHIKV-181/25-P2.P4 derivative strains were developed based on attenuating phenotypes previously described for alphavirus vaccines. The first of these derivatives, CHIKV-181/25-P2.P4.IRES, incorporates the internal ribosomal entry site (IRES) from encephalomyocarditis virus in place of the CHIKV 26S sub-genomic promoter and linker regions, offering a restrictive method for generating the structural elements of the virion at sub-optimal concentrations (Fig. 1c). The IRES prohibits CHIKV replication in insect cells, limiting the potential for vaccine circulation via vectors. The second derivative strain, CHIKV-181/25-P2.P4.E3/E1, was generated by removing the furin cleavage signal (Arg-Arg-Gln-Arg) in the pE2 glycoprotein required for complete processing into E3 and E2 and incorporating a compensatory mutation at E1^H253S^ homologous to that used for VEEV vaccine strain V3526 (Fig. 1d). Importantly, both attenuation strategies cannot be overcome by individual point mutations and represent a more significant hurdle for the LAV to revert to virulence.

We first sought to characterize the infection kinetics of CHIKV-181/25-P2.P4 and both IRES and E3/E1 derivatives in vitro. All variants were rescued from infectious clones in BHK-21 cells with titers ranging from 10^6^–10^7^ PFU/mL. At a multiplicity of infection (MOI) of 1, selected to assess near single-step kinetics, all variants demonstrated replication competence in both BHK-21 and Vero cells, an important production consideration for LAVs. Fidelity-variant CHIKV-181/25-P2.P4 produced similar titers as its parental strain in both baby hamster (BHK-21) and *Ae. albopictus* mosquito (C6/36) cells. An approximately threefold boost in infection efficiency was observed in BHK cells 12 h post inoculation in CHIKV-181/25-P2.P4, but titers were no different 24 or 48 h post-infection (Fig. 1e). As expected, both CHIKV-181/25-P2.P4.IRES and CHIKV-181/25-P2.P4.E3/E1 attenuated derivative strains infected cells at slower rates and achieved lower titers at 48 h (determined to be the peak in preliminary experiments) than their parent strain in BHK-21 cells (*P* < 0.0001) (Fig. 1e). CHIKV-181/25-P2.P4.IRES was unable to replicate in C6/36 cells, consistent with previous findings (Fig. 1f). The attenuating phenotype of CHIKV-181/25-P2.P4.E3/E1 also drastically reduced infection in C6/36, with the virus undetectable until 48 h post inoculation and even then only at low titer just above the limit of detection. These data demonstrate that the replication fidelity-altering mutations, nsP2^G641D^ and nsP4^C483Y^, do not drastically alter the peak infection titer of CHIKV-181/25 in vitro, but the IRES and E3/E1 derivatives are significantly attenuated compared to their progenitor while maintaining a level of infection competence necessary for propagation as LAV candidates.

### CHIKV LAV derivatives are avirulent in neonatal mice

**Fig. 2:**
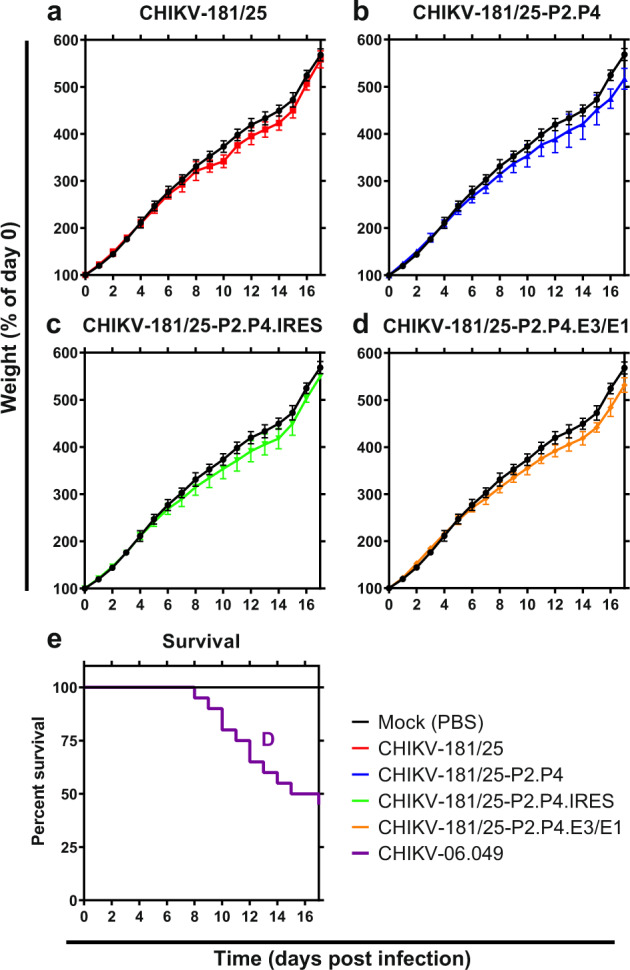
CHIKV live-attenuated vaccine derivatives are avirulent in neonatal mice. Two-day-old CD-1 IGS suckling mice were inoculated s.c. with 10^3^ PFU of each derivative CHIKV LAV or WT virus. Normalized weight (**a**–**d**) and mortality (**e**) were monitored for the duration of infection. *P* < 0.0001 (D), 2-way ANOVA (**a**–**d**), log-rank test (**e**), *n* = 9–10 per experiment, two combined experiments. Mean and standard deviation are indicated with data points and error bars (**a**–**d**).

Having established replication competence for each of our CHIKV-181/25 LAV derivatives, we next sought to demonstrate safety in a highly susceptible model of CHIKV infection. Outbred CD-1 IGS mice under 6 days of age are susceptible to lethal challenge with WT CHIKV, but administration of CHIKV-181/25 confers no discernible disease phenotype. We inoculated 2-day-old CD-1 IGS pups with 10^3^ PFU of each CHIKV LAV s.c. and monitored for weight and signs of neurological disease for 18 days. Animal weights for all vaccine groups tracked closely with mock-inoculated mice (*P* > 0.05) (Fig. 2a–d). Mortality was observed in WT CHIKV-06-049 challenged mice as early as day 8 and reached 50% by 18 days (Fig. 2e). No vaccine-treated animals showed signs of neurological disease or systemic inflammatory response syndrome and no mortality was observed.

### All fidelity-variant CHIKV LAVs except IRES derivative protect against virulent challenge

**Fig. 3:**
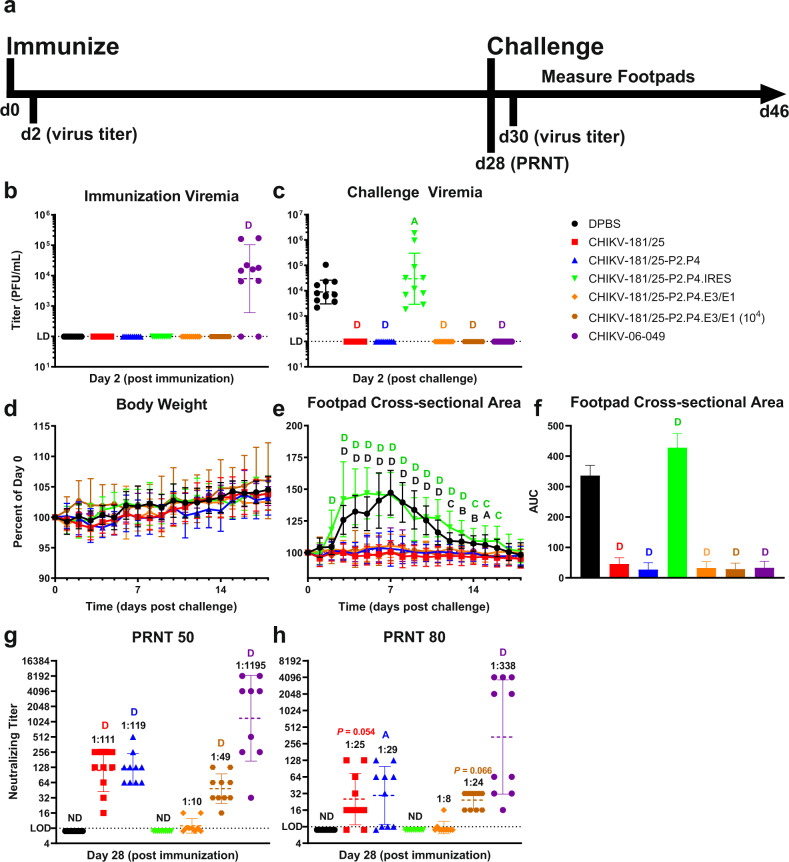
Fidelity-variant CHIKV live-attenuated vaccine generates protective immunity in mice. Adult C57Bl/6J mice were immunized with 10^3^ PFU CHIKV LAV derivatives then challenged bilaterally s.c. in footpads with 10^3^ PFU of WT CHIKV after 28 days. A timeline for infection and sample collection is shown (**a**). Peak viremias (**b**, **c**), weight change (**d**) hindlimb swelling (**e**), and total swelling area under the curve (**f**) are shown post immunization or challenge as indicated. Antibody titers are given for 50% (**g**) and 80% (**h**) neutralization thresholds on sera collected on day 28 prior to the challenge. *P* < 0.05 (A), 0.01 (B), 0.001 (C), 0.0001 (D), 2-way ANOVA on untransformed (**d**–**f**), and log-transformed (**b**, **c**, **g**–**h**) values, *n* = 5 per experiment, two combined experiments. Geometric mean and geometric standard deviation are indicated with lines and error bars (**b**–**d**, **g**–**h**). Mean and standard deviation are indicated with data points/bars and error bars (**d**–**f**). PRNT plaque reduction neutralization test, AUC area under the curve, ND not determined.

The virulent challenge of inbred adult mice with CHIKV, but not CHIKV-181/25, results in a discernible biphasic swelling phenotype lasting between 14 and 21 days, depending on age. We inoculated cohorts of 6-week-old C57Bl/6J mice s.c. in one rear footpad with 10^3^ PFU of each vaccine candidate and assessed peak viremia 2 dpi and monitored for signs of swelling indicating a loss of attenuation. The swelling was not observed in mice inoculated with experimental vaccine candidates, but typical biphasic swelling was present in WT CHIKV inoculated animals (Supplementary Fig. 1). Blood was collected 28 dpi to measure antibody production in serum and mice were subsequently challenged bilaterally with 10^3^ PFU WT CHIKV-06-049 s.c. in both rear footpads. CHIKV disease progression was measured by footpad cross-sectional area and peak viremia 2 days post-challenge. An outline of the study design is presented in Fig. 3a.

Following immunization, the LAV virus was undetectable in the blood 2 dpi (Fig. 3b). Viremia was not observed post-challenge in animals immunized with CHIKV-181/25, CHIKV-181/25-P2.P4, and CHIKV-181/25-P2.P4.E3/E1 (WT CHIKV-06-049 is shown for comparison). However, a more than 10-fold increase in the infectious virus was observed in mice administered CHIKV-181/25-P2.P4.IRES LAV versus unimmunized controls (*P* < 0.05) (Fig. 3c). Similarly, weight changes and footpad swelling after challenge were not observed in animals that received CHIKV-181/25, CHIKV-181/25-P2.P4, or CHIKV-181/25-P2.P4.E3/E1 LAVs (Fig. 3d, e). However, animals that received CHIKV-181/25-P2.P4.IRES developed significant swelling that peaked earlier and lasted longer than in unimmunized controls (*P* < 0.0001) (Fig. 3e). The cumulative post-challenge footpad swelling over time was significantly reduced for all immunized animals except those that received CHIKV-181/25-P2.P4.IRES, which experienced a ~25% increase in overall swelling for the duration of the experiment (*P* < 0.0001) (Fig. 3f).

Neutralizing antibody has been suggested as a correlate of vaccine protection against chikungunya disease^45^ and therefore serves as an important target for a LAV candidate. Serum from blood collected 28 days post-immunization was cross-neutralized with WT CHIKV-06-049 to assess plaque reduction. CHIKV-181/25 and CHIKV-181/25-P2.P4 produced geometric mean 50%-plaque neutralizing titers of 1:111 and 1:119, respectively (*P* < 0.0001) (Fig. 3g). At a matched immunization dosage of 10^3^ PFU, which was verified by back-titration of the inoculum, CHIKV-181/25-P2.P4.E3/E1 produced a reduced mean neutralizing titer of 1:10 and CHIKV-181/25-P2.P4.IRES antibody was undetectable (*P* > 0.05) (Fig. 3g). Increasing the immunization dosage of CHIKV-181/25-P2.P4.E3/E1 to 10^4^ PFU resulted in an increase in geometric mean 50%-neutralizing capacity from 1:10 to 1:49 (*P* < 0.0001) (Fig. 3g). Similar relative antibody titers were observed at the more stringent 80%-neutralization level (Fig. 3h). The ablation of viremia and absence of swelling correlated with the detection of neutralizing antibody. Due to the non-protective nature of CHIKV-181/25-P2.P4.IRES and inherent limitations on LAV viral yield for escalating the dosage, this LAV candidate was excluded from further consideration in our studies.

### Fidelity-variant CHIKV LAV restricts challenge virus replication in key tissues

**Fig. 4:**
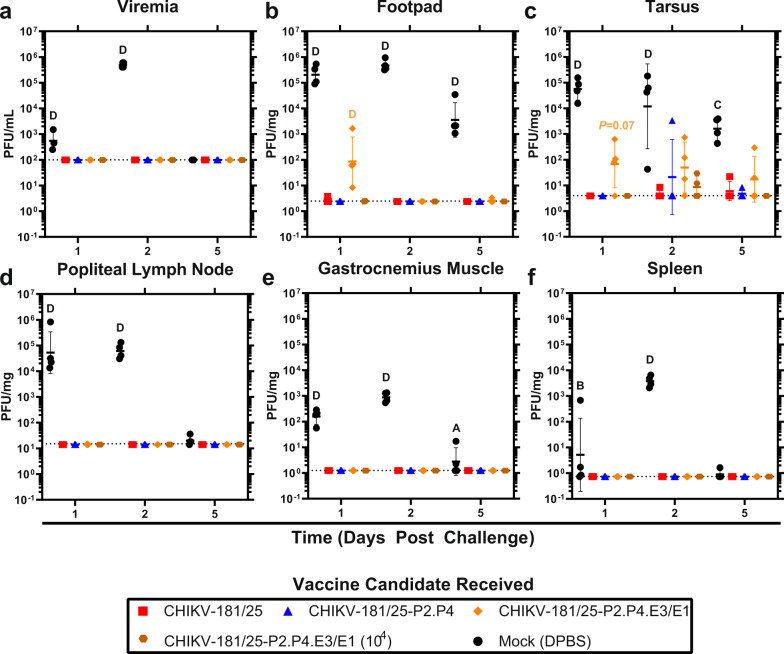
Immunization with a fidelity-variant vaccine restricts CHIKV dissemination. Adult C57Bl/6J mice were immunized with 10^3^ PFU (10^4^ PFU where indicated) of CHIKV LAV then challenged bilaterally s.c. in the footpad with 10^3^ PFU WT CHIKV on day 28. Animals were killed 1-, 2- and 5-days post-challenge, and viral load was measured in the blood (**a**) and selected replication-competent tissues (**b**–**f**). *P* < 0.05 (A), 0.01 (B), 0.001 (C), 0.0001 (D), 2-way ANOVA on log-transformed values, *n* = 4, one experiment. Geometric mean and geometric standard deviation are indicated with lines and error bars.

To better understand LAV-mediated CHIKV restriction, we examined WT CHIKV-06-049 replication in tissues both local to the site of infection (footpad, tarsus, muscle, lymph node) and systemic (blood, spleen) in immunized animals. CHIKV was detectible within 1 dpi and peaked at 2 dpi with infectious virus levels declining at 5 dpi (Fig. 4a–f). CHIKV-181/25 offered near sterilizing immunity in all tissues tested with fewer than 2 log_10_ PFU/mg tissue detected at any time point (Fig. 4a–f). CHIKV-181/25-P2.P4 was similarly protective over time, with detection of challenge virus in the tarsus of only one mouse at 2 dpi (*P* > 0.05) (Fig. 4c). 2 log_10_ PFU/mg of CHIKV-06-049 was detected in both the footpad and tarsus of animals vaccinated with CHIKV-181/25-P2.P4.E3/E1 at 10^3^ PFU at 1 dpi, and virus remained detectible in the tarsus through 5 dpi (Fig. 4b, c). At the higher dosage of 10^4^ PFU, CHIKV-181/25-P2.P4.E3/E1 was protective against viral replication in all tissues tested, comparable to CHIKV-181/25 and CHIKV-181/25-P2.P4 (Fig. 4a–f). These findings demonstrate that despite apparent protection from the disease at 10^3^ PFU, CHIKV-181/25-P2.P4.E3/E1 requires a higher inoculum than its parent to suppress WT CHIKV replication in tissues targeted by the virus to levels observed with CHIKV-181/25 immunization. Regardless of the presence or absence of inoculation site replication, the virus remained undetectable in the periphery in all immunized cohorts (Fig. 4a, d–f).

**Fig. 5:**
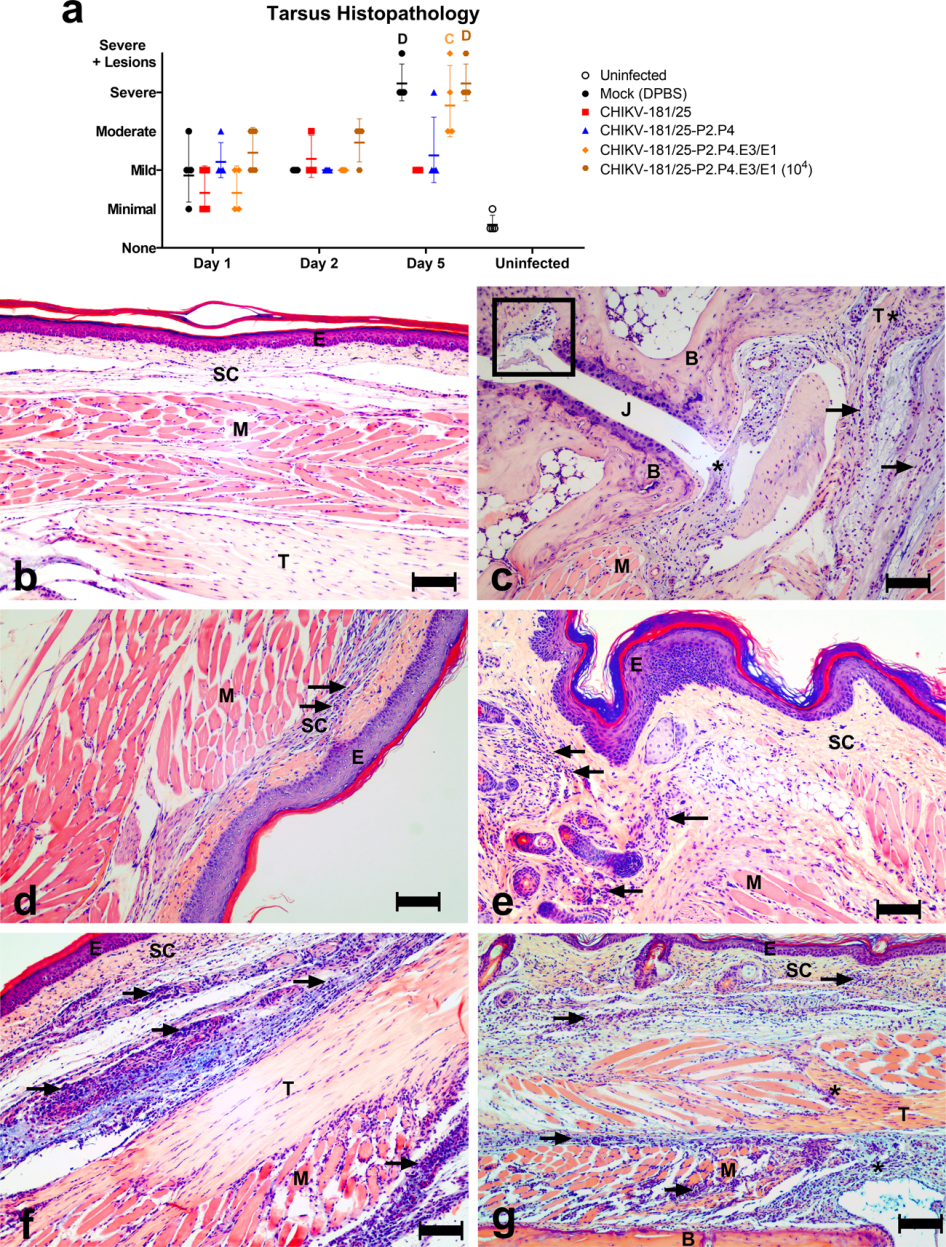
Fidelity-variant CHIKV vaccine protects mice from pathology associated with the virulent challenge. Hindlimbs from adult C57Bl/6J mice immunized with 10^3^ PFU (10^4^ PFU where indicated) CHIKV LAV and challenged bilaterally with 10^3^ PFU at day 28 with WT CHIKV were collected at days 1, 2, and 5 post-challenge and prepared for hematoxylin and eosin staining of thin sections. Cross-sections were blindly evaluated by a veterinary pathologist and scored according to Supplementary Table 2. Composite group scores are given for each time point (**a**) and representative images are shown for each group at 5-days post-challenge. DPBS only (**b**) DPBS + CHIKV WT (**c**) CHIKV-181/25 (**d**) CHIKV-181/25-P2.P4 (**e**) CHIKV-181/25-P2.P4.E3/E1 (**f**), and CHIKV-181/25-P2.P4.E3/E1 (10^4^ PFU) (**g**). Arrows highlight inflammatory infiltrates. The rectangle indicates inflammatory cells and debris within a metatarsal joint space (**c**). Asterisks indicate inflammatory cell infiltrates within the synovial membrane (**c**) and tendon sheath (**f–g**). Mean and standard deviation are indicated with lines and error bars (**a**). E epithelium, SC subcutaneous tissue, M muscle, B bone, T tendon, J joint, bar = 100 µm. *P* < 0.001 (C) 0.0001 (D), 2-way ANOVA, *n* = 4/group, one experiment.

Hindlimbs from each group were evaluated for CHIKV-induced histopathologic lesions. By day 5 post-challenge, histopathology scores for CHIKV-181/25 and CHIKV-181/25-P2.P4 were significantly lower than mock-infected controls, which is consistent with the viral titers measured in the same animals (Fig. 4a–f). However, mice vaccinated with both doses of CHIKV-181/25-P2.P4.E3/E1 had consistently higher scores, similar to mock-vaccinated controls (Fig. 5a). The most common histopathologic finding (Fig. 5b–g) following challenge was lymphoplasmacytic and histiocytic to neutrophilic inflammation of the subcutaneous tissue, fascia, and/or skeletal muscle (myositis), with moderate to severe edema. There were variable myocyte degeneration and necrosis, scattered tendonitis, and occasional inflammatory infiltrates expanding the synovial membrane. In one mock-vaccinated mouse, there were few inflammatory cells and rare necrotic debris within a metatarsal joint space. Overall, the severity of tarsal/metatarsal joint pathology varied with vaccine candidate (with the most severe lesions in mock-vaccinated controls and those vaccinated with CHIKV-181/25-P2.P4.E3/E1) and was most severe by 5 days after challenge.

### Fidelity-variant LAVs accumulate greater population diversity than CHIKV-181/25

**Fig. 6:**
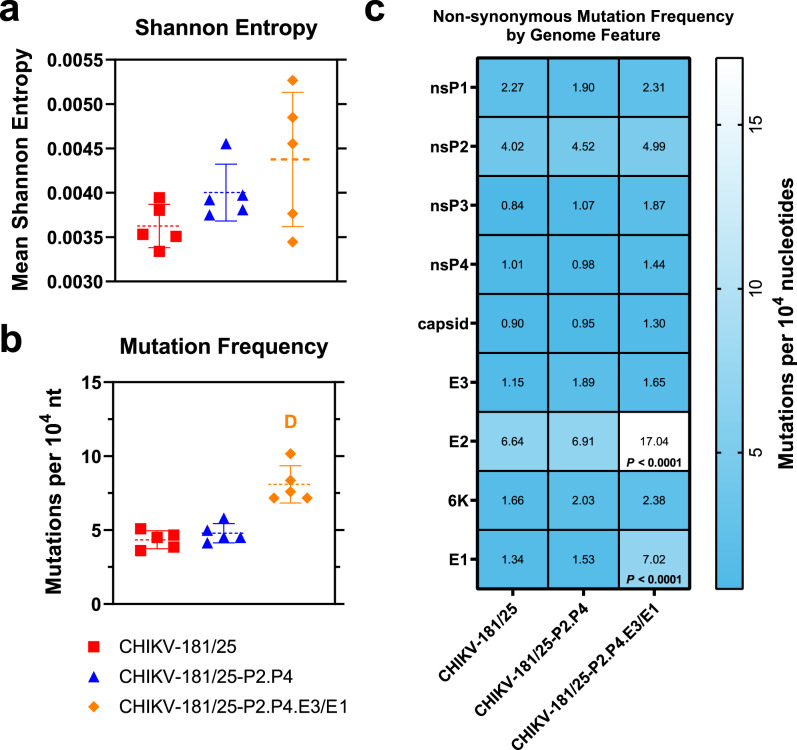
Apparent mutational accumulation following serial passage is unaffected by replicase fidelity-modulating variant. CHIKV LAV candidates were serially passaged at MOI = 0.1 in BHK cells and the passage-10 virus was deep-sequenced. Reads were aligned to the consensus genome. Statistics including Shannon entropy (**a**) mutation frequency (**b**) and non-synonymous mutation frequency (**c**) were calculated with ViVan^61^. *P* < 0.0001 (D), ANOVA (**a**, **b**), 2-way ANOVA (**c**), *n* = 5, one experiment. Mean and standard deviation are indicated by lines and error bars (**a**, **b**).

Although the population genetic diversity of the nsP2^G641D^/nsP4^C483Y^ double replicase mutant was previously described for WT CHIKV^26,30^, to measure the effect of these mutations on LAV diversification in the CHIKV-181/25 background used in these studies, each candidate was serially passaged ten times in BHK-21 cells in five independent replicates at an MOI = 0.1 to avoid population bottlenecking. Resultant passage-10 viruses were deep-sequenced and aligned to the consensus genome sequence of the unpassaged progenitor stock virus to measure accumulated variation in each LAV. A python-based viral variance analysis tool, ViVan, was used to compare mutation frequency and accumulation between passaged candidates. Shannon entropy, a measure of unexpectedness used here to describe average single-nucleotide polymorphisms across the full CHIKV genome, revealed no significant differences in variance between CHIKV-181/25 and either CHIKV-181/25-P2.P4 or CHIKV-181/25-P2.P4.E3/E1 (*P* > 0.05) (Fig. 6a). However, there is a trend toward increasing Shannon entropy in the fidelity-variant candidates compared to CHIKV-181/25, consistent with previous findings published by our lab^30^. The overall frequency of mutations between CHIKV-181/25 and CHIKV-181/25-P2.P4 is similar, with a small, nonsignificant increase in variant accumulation in CHIKV-181/25-P2.P4 (*P* > 0.05) (Fig. 6b). In contrast, CHIKV-181/25-P2.P4.E3/E1 accumulated nearly twice as many single-nucleotide polymorphisms as CHIKV-181/25 by passage-10 (*P* < 0.0001) (Fig. 6b), owing to hypermutation of the E2 glycoprotein in the N-terminal region proximal to the removed furin cleavage site and high mutational accumulation in the E1 glycoprotein (Supplementary Fig. 2). These changes are also reflected in an increase in non-synonymous mutations resulting in observed amino acid variation in CHIKV-181/25-P2.P4.E3/E1 versus CHIKV-181/25 (*P* < 0.0001) (Fig. 6c). None of the passaged fidelity-variant LAV candidates experienced consensus reversion at the nsP2, nsP4, or E1 engineered point mutant loci (Supplementary Fig. 3). Also of note, there was no reversion of the previously described attenuating loci E2^I12^ or E2^R82^ ^22^ in any replicate by passage-10, including the parental CHIKV-181/25.

### Diversification of CHIKV-181/25-derived LAV is associated with virulent reversion

Having established apparent mutation frequencies of each LAV, we next sought to determine whether individual candidates exhibited a greater propensity to revert to virulence in an animal model of CHIKV disease. To increase yield from an immune-privileged tissue, each LAV candidate was triplicate passaged i.c. in suckling mice five times to facilitate host adaptation. The resulting passage-5 LAVs were inoculated i.c. in parallel to their unpassaged progenitors in 2-day old CD-1 IGS mouse pups and monitored for signs of CHIKV disease. CHIKV-181/25-P2.P4.E3/E1 was unrecoverable after passaging once in the suckling mouse brain at multiple tested time points (48 h p.i., *n* = 10, 72 h p.i., *n* = 2). For this reason, CHIKV-181/25-P2.P4.E3/E1 was unable to be serially passaged five times and was excluded from subsequent analysis.

**Fig. 7:**
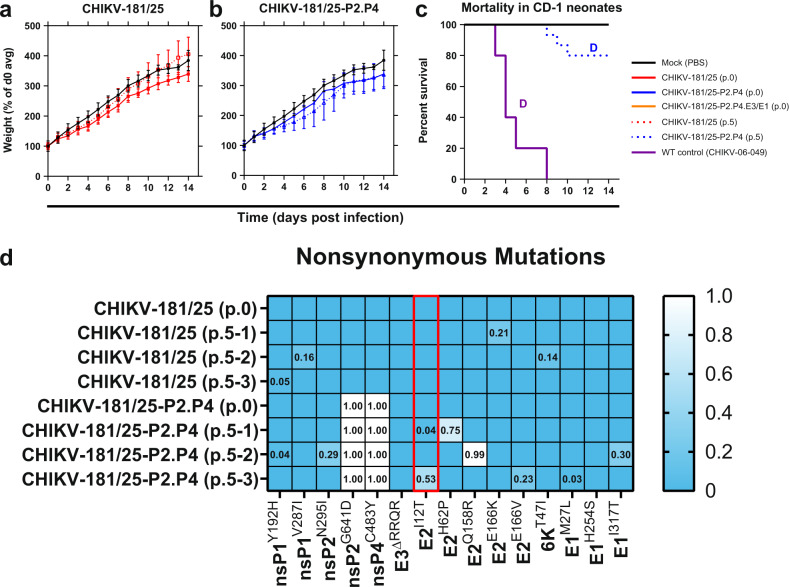
Fidelity-variant CHIKV live-attenuated vaccine demonstrates reversion potential in vivo. LAV candidates were passaged 5 times in triplicate at 10^4^ PFU in the brains of 6-day old suckling CD-1 mice. Three combined replicate passaged and single unpassaged LAVs were inoculated i.c. in 2-day old CD-1 mice and weight (**a**–**b**) and survival (**c**) were monitored. Viruses were sequenced using PrimalSeq and non-synonymous mutations were identified with iVar (**d**). *P* < 0.0001 (D), log-rank test, n = 15, 1 experiment. Mean and standard deviation are indicated with data points and error bars (**a**–**b**). Passage number and biological replicate are indicated in parentheses for individual samples. The red box indicates a known virulent reversion.

Similar to the s.c. inoculation route, i.c. inoculation of unpassaged CHIKV-181/25 and CHIKV-181/25.P2.P4 LAVs caused a slight reduction in weight gain compared to mock-inoculated animals (*P* > 0.05) (Fig. 7a–b) and resulted in no observed disease. Mice inoculated with passage-5 CHIKV-181/25 exhibited a similar delay in weight gain that recovered by 14 dpi (Fig. 7a). One replicate (out of 3) of CHIKV-181/25-P2.P4 resulted in lethality in a subset of animals receiving passage-5 virus with the surviving cohort experiencing notable weight loss (Fig. 7b, c) and lasting hindlimb paresis even after weight stabilized. To probe the variance in these LAV replicates that produced disparate disease outcomes in suckling mice, both unpassaged and mouse brain passage-5 LAVs were sequenced using PrimalSeq with custom primers designed in PrimalScheme (Supplementary Table 1) and analyzed with iVar^46,47^. Non-synonymous mutations were observed in every passaged LAV, with 4 identified in CHIKV-181/25 and 8 arising in CHIKV-181/25-P2.P4 (Fig. 7d). Notably, a reversion to the WT sequence was observed at CHIKV-181/25-P2.P4 E2^I12^ in two of three replicates, including at 53% in the replicate where neonate mortality was observed. These data show that modification of the CHIKV-181/25 LAV by adding 2 replicase mutations is not sufficient to eliminate virulent reversion in suckling mice.

### CHIKV fidelity-variant LAV does not alter *Ae. aegypti* mosquito infectivity

One complication to the development of a replication-competent vaccine for a vector-borne disease is the potential for vaccine transmission via vectors. To ensure that fidelity-variant CHIKV LAVs are not more transmissible than WT CHIKV or CHIKV-181/25 in the primary mosquito vector, *Ae. aegypti*, we exposed naive mosquitoes to infectious bloodmeals and measured infection rates ascertained from mosquito bodies and transmission rates in saliva at 10 days post bloodfeeding. Due to the lower production yield of CHIKV-181/25-P2.P4.E3/E1, a 2-log reduction in the infectious virus was presented in mosquito bloodmeals compared to the other vaccine candidates tested (10^5^ PFU/mL versus 10^7^ PFU/mL)—a significant caveat that must be considered when comparing these viruses. Mosquitoes demonstrated similar infection rates by CHIKV-181/25, CHIKV-181/25-P2.P4, and CHIKV-181/25-P2.P4.E3/E1 as measured by both infectious virus (23%, 30%, and 10%, respectively, *P* > 0.05) and RNA (37%, 37%, and 20%, respectively, *P* > 0.05) in bodies (Table 1). Similarly, no significant increase in transmissibility was observed for fidelity-variant LAV compared to CHIKV-181/25 (0%, 3%, and 0%, respectively, for both infectious virus and RNA, *P* > 0.05), indicating that the replicase fidelity mutations offer no fitness benefit to LAV infection of and transmission by the primary vector.

**Table 1:** CHIKV-181/25-derived LAVs are not efficiently transmitted by *Ae. aegypti*.

Virus	Bloodmeal titer (PFU/mL)	Infected (infectious CHIKV)	Transmitting (infectious CHIKV)	Infected (CHIKV RNA)	Transmitting (CHIKV RNA)
CHIKV-181/25	6.5 × 10^6^	23% (7/30)	0% (0/30)	37% (11/30)	0% (0/30)
CHIKV-181/25-P2.P4	1.6 × 10^7^	30% (9/30)	3% (1/30)	37% (11/30)	3% (1/30)
CHIKV-181/25-P2.P4.E3/E1	1.8 × 10^5^	10% (3/30)	0% (0/30)	20% (6/30)	0% (0/30)
CHIKV-06-049	4.5 × 10^7^	30% (7/23)	9% (2/23)	44% (10/23)	22%^C^ (5/23)

*Ae. aegypti* mosquitoes colonized from Los Angeles, California, USA, were presented bloodmeals containing LAVs at titers reported. Saliva was collected to assess transmission rates and whole bodies were assayed for infection rate assessments 10 days post blood feeding. Infectious CHIKV and RNA were determined by plaque assay and qRT-PCR, respectively. Table values are percentage (positive/total). *P* < 0.001 (C) Chi-square test, *n* = 23–30, 1 experiment.

## Discussion

Replication-competent vaccines provide robust protection with a single dose and are remarkably scalable. Live-attenuated viruses such as the yellow fever virus strain 17D produce strong cellular and humoral immunity that can last 10 or more years in healthy adults with no need for an adjuvant or prime-boost schedule^48^. This potent level of protection comes at a cost, with potential lot-to-lot variation^49^ and intrahost mutation during LAV replication that can lead to virulent phenotypes in vaccinees with underlying conditions and even healthy adults^50–52^. Complicating matters, RNA-dependent RNA polymerases lack proofreading capabilities and incorporate erroneous nucleotides in progeny genomes at relatively higher rates than DNA-genomic counterparts^24,25^, resulting in a higher rate of genetic drift, which is undesirable in an attenuated pathogen for commercial propagation as a vaccine. Two ways to circumvent this challenge are to either incorporate attenuating elements in a LAV that cannot be easily reverted through point mutation or recombination, similar to approaches currently in use for a novel poliovirus LAV^34^ or to reduce the error rate of the attenuated pathogen, thus reducing the reversion potential. In this study, we explored both approaches with an emphasis on designing a high-fidelity-variant of a CHIKV LAV known to be genetically unstable during human clinical testing.

All vaccine candidates generated for this study include two genetic variants identified in an Indian Ocean Lineage CHIKV strain 06–049 following replicate serial passage in the presence of either of two nucleoside analogs, ribavirin and 5-fluorouracil^26^. While nsP2^G641D^ has been demonstrated to help CHIKV resist the effects of nucleotide pool depletion^53^, the mechanism(s) of mutagen resistance for nsP4^C483Y^ has not been resolved. However, the combination of both variants was demonstrated to reduce erroneous nucleotide selection by up to 50%, even in the absence of a nucleoside analog driving hypermutation^26^. While the observed decrease is notable, fidelity-variant CHIKV accumulated mutations at a frequency of >1 per 10^4^ nucleotides, leaving significant room for virulent reversion^26,53^. A follow-up study from our laboratory using next-generation sequencing (NGS) to further elucidate the effects of these two loci on replication fidelity demonstrated an unexpected increase in mutation frequencies with either the nsP4^C483Y^ mutation alone or in combination with nsP2^G641D ^^30^. However, a mechanism for this discrepancy between fidelity phenotypes measured by Sanger sequencing versus NGS remains elusive, biases resulting from oxidative damage during NGS library preparation have been ruled out as an underlying cause^30^. This disparity in fidelity phenotype as measured by NGS was also observed in the present study with CHIKV-181/25, although less pronounced: no single measure of error frequency for the replicase mutant LAV demonstrated a significant increase versus the parental LAV strain. While mutations observed were largely non-coding in nature (either synonymous mutations in the coding region or mutations in the UTRs), several notable non-synonymous mutations were observed in the CHIKV-181/25-P2.P4 fidelity-variant after passage in both cell culture and mice. Notably, a known virulent reversion, E2^I12T^, arose in CHIKV-181/25-P2.P4 passaged in mice, which was not present in the CHIKV-181/25 parental strain after passaging under the same conditions. These results strongly suggest that the trend of increasing mutation accumulation has a significant impact on the potential for virulent reversion by our fidelity-variant LAV. Complicating matters, RNA virus mutant spectra arising from mutational accumulation during replication have been postulated to complement functions during productive infection, leading to virulent phenotypes. Indeed, vaccines like yellow fever 17D-204 have been shown to have a highly restricted spectrum of mutant RNAs compared to their parent WT virus, which is thought to play an important role in the maintenance of attenuation^51^. Similarly, poliovirus with a limited mutant repertoire is attenuated compared to its parent, suggesting a pathogenic role for mutational sampling by an erroneous viral replicase^54^. In this context, the trend toward increased mutation frequency by CHIKV-181/25-P2.P4 also raises the possibility that a more diverse virus population may either contribute to vaccine virulence or decrease lot-to-lot uniformity of a LAV incorporating these mutations.

Despite the reversion potential, our fidelity-variant vaccine candidates, CHIKV-181/25-P2.P4 and CHIKV-181/25-P2.P4.E3/E1, were well tolerated in multiple susceptible mouse models of infection and produced no signs of the disease while generating a robust immune response, including a detectible neutralizing antibody titer at or above levels observed during subclinical human infection^55^. The degree of protection was dose-dependent, with CHIKV-181/25-P2.P4.E3/E1 requiring a 10-fold greater inoculum than either CHIKV-181/25-P2.P4 or the parental CHIKV-181/25 LAV to achieve similar antibody titers. Likewise, immunized mice were protected from ipsilateral footpad swelling and produced no detectible serum viremia, but challenge virus restriction in individual tissues required a 10-fold increase in CHIKV-181/25-P2.P4.E3/E1 inoculum to achieve similar protection. Similarly, mild to moderate mononuclear cell infiltration was observed in the superficial fascia between 2- and 5- days post-challenge, but no significant joint or tendon lesions were observed. Together, our results demonstrate that both CHIKV-181/25-P2.P4 and CHIKV-181/25-P2.P4.E3/E1 are capable of generating protective immunity. Importantly, the replicase fidelity-altering mutations explored in this study do not increase morbidity or mortality associated with the unpassaged CHIKV-181/25 LAV in mice and have no significant effect on virus fitness in *Ae. aegypti* mosquitoes, the primary vector.

It must be stressed, however, that vector competence of the LAVs explored in this study comes with the caveat that CHIKV-181/25-P2.P4.E3/E1 was presented at nearly 2-logs lower concentration due to limitations on LAV recovery yield in vitro. While this challenge prevents us from definitively saying that CHIKV-181/25-P2.P4-E3/E1 is non-transmissible in mosquitoes, the lack of a significant increase in CHIKV-181/25-P2.P4 vector competence is promising nonetheless. Furthermore, vaccine-induced viremia is required for mosquito vectors to become infected with a LAV, and sufficiently high titers must be present to facilitate transmission. While extremely rare, LAV VEEV used in equines on the Texas Gulf Coast in 1971 was detected in local mosquito populations in Louisiana, USA where the LAV was not administered^56^, underscoring the need to restrict vector competence.

Unexpectedly, one derivative vaccine based on the CHIKV-181/25-P2.P4 candidate failed to elicit adequate protection in our studies. CHIKV-181/25-P2.P4.IRES, which is based on attenuation of an Indian Ocean Lineage CHIKV strain, proved challenging to produce in sufficient quantities for immunization and was non-protective at the available dosage. This candidate produced lower peak titers in vertebrate cells and failed to infect mosquito cells and was well tolerated in neonatal mice. However, immunization with CHIKV-181/25-P2.P4.IRES failed to produce a measurable neutralizing antibody response and pre-exposure was associated with a reproducible increase in disease severity following challenge with WT CHIKV. We propose that poor vaccine replication in mice resulting from reduced structural protein production, which was four- to six times lower than the WT virus for the related alphavirus, VEEV^41^, combined with the attenuation of CHIKV-181/25 was insufficient to trigger the production of adequate immunity including neutralizing antibodies. This is in stark contrast to the CHIKV-IRES vaccine raised in the WT background, which was both protective and yielded sufficient viral titers for vaccination studies^35–37^. It is possible that weak immunological memory associated with CHIKV-181/25-P2.P4.IRES vaccination is directly responsible for this increase in disease severity. In contrast, our other derivative vaccine that incorporated a pE2-cleavage defect, CHIKV-181/25-P2.P4.E3/E1, was similarly well-tolerated and generated viable antibody responses, both protecting immunized mice from measurable footpad swelling and viremia. No tested LAV, including CHIKV-181/25, produced sterilizing immunity, with challenge virus detectable in either the footpad or tarsus joint post infection. Our histological findings similarly confirm that all vaccinated animals experienced mild to moderate cellular infiltration post-challenge, but lacked the bone and tendon pathologies associated with CHIKV infection in naive animals.

Counter to our original goal of generating a high-fidelity CHIKV LAV, CHIKV-181/25-P2.P4.E3/E1, which produced the highest mutation frequency following cell culture passage, was nevertheless well-tolerated, immunogenic, and failed to propagate long enough to produce reversion mutations in vivo. As anticipated, a strong selective pressure was observed, resulting in hypermutation of regions proximal to the deleted furin cleavage signal as was observed in replicate passages of CHIKV-181/25-P2.P4.E3/E1. The cumulative instability of this LAV resulted in poor replication in both cells and mouse brains, the latter of which resulted in non-viable titers for serial passaging. Presumably, this poor replication capacity is the reason a higher inoculum is required to achieve protective immunity than other tested candidates. However, the self-limiting infection also significantly decreases the potential for virulent reversion, a key target for safety trials of any LAV. Still, hypermutation is concerning for the clinical evaluation of a LAV, and further study of the E3^ΔRRQR^/E1^H253S^ mutations alone or in combination with fidelity-variant CHIKV-181/25 is necessary before considering this approach.

While we demonstrated that the fidelity-variant mutations, nsP2^G641D^ and nsP4^C483Y^, are not sufficient on their own to increase the safety of CHIKV-181/25 in mice, they do not negatively affect the robust protection from CHIKV disease conferred by CHIKV-181/25 in mice. These mutations are stably incorporated and are not lost over successive rounds of LAV replication, and in combination with the selective pressure of a pE2-cleavage defect, provide a self-limiting hypermutation effect while still providing protective immunity. We highlight an alternative strategy to our proposed goal of increasing virus fidelity to preserve LAV safety and instead strike a balance between attenuation and replication competence. Importantly, the role of hypermutation in the attenuated phenotype of CHIKV-181/25-P2.P4.E3/E1 requires further study. Our work also builds on a growing body of evidence that the accurate assignment of replicase fidelity requires direct mutation rate measurements in contrast to the snapshot of mutational accumulation provided by indirect sequencing methods^29,30,57^. Before CHIKV-181/25-P2.P4.E3/E1 can be fully evaluated as a viable vaccine candidate, further efforts are required to directly probe replication errors associated with CHIKV polymerase mutants, similar to related studies for influenza and VEEV^57,58^.

## Methods

### Mice

Pregnant CD-1 IGS mice from Charles River Laboratories were received at 15 days gestation and housed individually. Litters were pooled at the time of birth and evenly redistributed to surrogate dams for subsequent virulence testing or serial passage. 6-week old C57Bl/6J male and female mice were received from Jackson Laboratories for immunization and challenge experiments. Mice were housed in ABSL-3 conditions at 22–25 °C, 12 h light: 12 h dark cycle with rodent chow, and sterile water ad libitum for the duration of experiments. Mice were co-housed with 2–4 animals of the same sex and treatment group per cage.

### Ethics statement

Mouse studies received ethical approval by the Institutional Animal Care and Use Committee for the University of California, Davis, under protocol #20966. The University of California, Davis is an Association for Assessment and Accreditation of Laboratory Animal Care (AAALAC) accredited institution.

### Cell culture

BHK-21 [clone 13] (ATCC CCL-10) and Vero (ATCC CCL-81) cells were maintained in Dulbecco’s Modified Eagle’s Medium (DMEM) supplemented with 10% Fetal Bovine Serum (FBS), 100 U/mL penicillin, and 100 μg/mL streptomycin at 37 °C and 5% CO_2_ in a humidity-controlled incubator. *Aedes albopictus* clone C6/36 (ATCC CRL-1660) cells were maintained in Schneider’s Insect Medium with 20% FBS at 28 °C and 5% ambient CO_2_ in a humidity-controlled incubator. Mammalian cells were passaged by trypsinization while adherent insect cells were scraped into suspension for subsequent passage.

### Viruses

A wild-type (WT) CHIKV-06.049 infectious clone isolated from a patient in La Réunion was obtained^59,60^. Infectious cDNA clones of CHIKV-181/25 and CHIKV-IRES were generously provided by Dr. Scott Weaver at the University of Texas Medical Branch^21,35^. The nsP2^G641D^ and nsP4^C483Y^ substitutions were introduced by site-directed mutagenesis (QuikChange Lightning multi site-directed mutagenesis kit; Agilent) into the cDNA clone of CHIKV-181/25 with a single point mutation in nsP2 (GGC > GAC) and nsP4 (TGC > TAC) to generate CHIKV-181/25-P2P4. The single-nucleotide changes that mutated the amino acid were selected since they arose experimentally during mutagen treatment^26^ and also to avoid stochastic effects of that could arise from changing RNA secondary structures by mutating more nucleotides. The CHIKV-181/25-P2P4.IRES cDNA clone was created by ligation of 12.5 kb *Sph*I-digested CHIKV-181/25-P2P4 and a 1925 bp *Sph*I-digested overlapping PCR product, which was generated by overlapping 3 individual PCR products of IRES (PCR1) amplified from CHIKV-IRES cDNA clones with 5′-CAGATCCAACTTCGAGAAGCTCAGAG and 5′-GGTCGAGGCTGGTACCTCCTATTG, the 3′-end of nsP4 (PCR2) with 5′-TGGAAGATCGTCTGACAAAATCCG and 5′-CTCTGAGCTTCTCGAAGTTGGATCTG, and the 5′-end of capsid gene (PCR3) with 5′-TACAATAGGAGGTACCAGCCTCGACC, and 5′-GGGGAGAACATGTTAAGGATGCTTG from CHIKV-181/25-P2P4. The CHIKV-181/25-P2P4.E3/E1 cDNA clone was generated by deleting four amino acids at the 3′ end of E3 (Δ60–63) to eliminate the structural polyprotein PE2-cleavage site and two nucleotide substitutions in E1 H251S (CAC > AGC) by site-directed mutagenesis. Genotypic integrity of all cDNA clones was verified by whole-genome Sanger sequencing. Infectious CHIKV was rescued from cDNA clones by plasmid linearization with *Not*I restriction endonuclease, in vitro transcription with the mMessage mMachine SP6 transcription kit (ThermoFisher), and electroporation of in vitro transcribed RNA in BHK-21 cells^30^. For rescued virus stocks and experiments described below, infectious virions were stored at −80 °C and thawed once for titration by Vero plaque assay.

### Plaque assays

Vero cells were seeded to confluence on 12-well cluster plates then incubated in duplicate with 100 µL of each serially diluted inoculum in phosphate-buffered saline (PBS) with 1% FBS for 1 h. After 1 h, cells were overlaid with 0.5% agarose in DMEM with 5% FBS, 100 U/mL penicillin, and 100 μg/mL streptomycin. Cells were incubated 72–96 h. Cells were fixed in 4% formaldehyde and agarose plugs were removed. Plaques were stained for 10 min with 0.05% w/v crystal violet in 20% ethanol then washed in cold water. Plates were dried inverted for at least 24 h prior to counting plaques. Virus titer was measured by manually counting adjacent technical duplicate wells of a dilution producing between 20–200 plaques and calculating the average.

### qRT-PCR

Total RNA was extracted from mosquito bodies or saliva, and suckling mouse brain using MagMax viral RNA isolation kit (ThermoFisher). RNA from serial brain-passage experiments was further depleted of rRNA using a human/mouse/rat rRNA depletion kit (New England Biolabs). One-step qRT-PCR was performed on 5 µL of RNA using TaqMan Fast Virus 1-step master mix (ThermoFisher), forward primer 5′-TCACTCCCTGTTGGACTTGATAGA-3′, reverse primer 5′-TTGACGAACAGAGTTAGGAACATACC-3′, and 5′-FAM-conjugated and 3′-Black Hole Quencher-1-conjugated 5′-AGGTACGCGCTTCAAGTTCGGCG-3′ TaqMan probe on a ViiA-7 qPCR thermal cycler (Applied Biosystems). Absolute quantification was performed with a standard curve of in vitro transcribed full-length CHIKV WT genomic RNA.

### Virus growth kinetics

BHK-21 and C6/36 were grown to 80% confluence in 24-well cell culture treated cluster plates (Corning) and inoculated with CHIKV variants at a multiplicity of infection equal to 1. Cells were incubated with the virus for 1 h at 37 °C then inoculum was aspirated, and cells were washed 4 times in sterile DPBS before replacing with fresh cell culture media. The supernatant was sampled for the first 48 h of infection then diluted and frozen at −80 °C in virus diluent consisting of DPBS without calcium and magnesium and 1% FBS. Viral replication in the collected supernatant was assessed by Vero plaque assay as described.

### Serial passage in BHK-21 cells

Five replicates of each vaccine candidate were seeded on confluent T-25 flasks of BHK cells at a multiplicity of infection (MOI) of 0.1 and expanded for 48 h. The recovered virus was titrated by Vero plaque assay and re-seeded at MOI = 0.1 on BHK-21 cells. Five replicate series for each vaccine candidate was repeated for 10 serial passages.

### Serial passage in suckling mouse brain

Six-day-old CD-1 suckling mice were inoculated intracranially (i.c.) with 10^4^ PFU of each virus diluted in sterile DPBS. Mice were housed for 48 h post-inoculation then killed. Brains were manually homogenized in 1 μL DMEM per mg tissue, then centrifuged to remove solid debris. Passaged virus was titrated on Vero cells and then used for the subsequent passage. After 5 serial passages, 3-day old CD-1 suckling mice were inoculated i.c. with 10^3^ PFU of each passaged virus or progenitor unpassaged virus, then monitored for weight loss and mortality.

### Next-generation sequencing

Four mL of cell culture-passaged viruses were concentrated to 100 µL in Amicon Ultra-4 regenerated cellulose membrane centrifugal filters for ≥10 kDa proteins (Millipore Sigma). Total RNA from retained supernatant or 50 µL of the brain-passaged virus was extracted using the MagMax viral RNA Isolation Kit (ThermoFisher). Brain-passaged viruses were also depleted of ribosomal RNA using the NEBNext rRNA Depletion Kit for Human/Mouse/Rat (New England Biolabs). Libraries for cell culture-passaged viruses were generated for Illumina sequencing using NEBNext Ultra II RNA Library Prep Kit for Illumina and NEBNext Multiplex Oligos Dual Index Primers (New England Biolabs) using 500 ng of input RNA. Cell culture libraries were sequenced using a 150 bp paired-end protocol on HiSeq 4000 (Illumina) for a target of >5000× genome coverage. Demultiplexed sequences were quality trimmed and aligned to the corresponding strain genome and variants were called using the Python-based virus variant analysis pipeline, ViVan^61^. Population diversity was assessed by ViVan as the mean nucleotide substitution frequency across genomic regions, and Shannon entropy. Brain-passaged viruses were processed using amplicon-based Illumina deep sequencing following the PrimalSeq pipeline due to the low quantity of input virus^46^. Primers tiling ~400 bp amplicons across the CHIKV-181/25 genome (Supplementary Table 1) were generated with PrimalScheme^47^ and validated for sequencing depth and coverage on 10^6^ genome copies of input virus. Paired-end 250 reads from unpassaged and suckling mouse brain passage-5 LAV candidates were sequenced using the Illumina MiSeq platform and assessed for variants called over 3% using iVar^46^.

### Mouse infections

#### Suckling mice

Two-day-old suckling CD-1 IGS mice were inoculated s.c. with 10^3^ PFU of each unpassaged CHIKV vaccine candidate or WT CHIKV-06-049. Inocula were back-titrated and verified to be within 0.5-log_10_ PFU of the target dosage. Similarly, passaged vaccine candidates (p5) from suckling mouse brain and their unpassaged progenitors were inoculated i.c. in 2-day-old CD-1 suckling mice at 10^3^ PFU in parallel cohorts to assess changes in virulence following brain passage. All mice were monitored daily for 17 days for weight changes and signs of neonatal CHIKV disease, including systemic inflammatory response syndrome, ataxia, and paralysis.

#### Adult mice

Six-week-old C57Bl/6J mice were inoculated subcutaneously (s.c.) in the left rear footpad with 103 PFU of vaccine candidates, WT CHIKV-06-049, or DPBS. Animals were monitored daily for weight change and the footpad cross-sectional area was recorded for 18 days post-inoculation (dpi). The cross-sectional area was calculated as an ellipse on the coronal plane by measuring the height and width of the footpad with calipers. Blood was collected via submandibular bleed and clotted for 10 min at room temperature then centrifuged for 15 min at 10,000 × *g* to separate the serum from the resulting clot. Serum from days 2 and 28 post-immunization was used to assess viremia and peak antibody titer, respectively. On day 28, animals were challenged s.c. with 103 PFU of WT CHIKV-06-049 in both rear footpads and monitored for weight and footpad cross-sectional area for 18 days. Mice were bled and serum was processed as described above on day 30, challenge day 2, to assess peak viremia following challenge. Additional vaccinated cohorts were serially killed and manually perfused with ice-cold sterile DPBS using a 20 mL syringe at days 1, 2, and 5 post-challenge to remove circulating virus in order to assess tissue virus levels in footpad dermis/fascia, tarsus, gastrocnemius muscle, popliteal lymph node, and spleen, which were enumerated by plaque assay. Hindlimbs were evaluated for the presence of CHIKV-induced histopathologic lesions. Hindlimb tissues (excluding femur) were fixed for 24 h in 10% neutral-buffered formalin, decalcified over 10 days in 0.5 M Ethylenediaminetetraacetic acid (EDTA) (pH = 7.2) and paraffin-embedded. A 5 µm thick section containing the entire hindlimb from 4 mice per group at each time point were stained with hematoxylin and eosin (H&E), blindly evaluated by a board-certified veterinary anatomic pathologist and scored according to the rubric shown in Supplementary Table 2.

### Plaque reduction neutralization tests (PRNT)

Serum from day 28 post immunization was heated to 56 °C for 30 min then diluted 1:4 in DPBS containing 1% heat-inactivated FBS (virus diluent). Serum was further serially twofold diluted in virus diluent, with the final well reserved for virus diluent only. WT CHIKV-06-049 was diluted to 800 PFU/mL in virus diluent and combined 1:1 with serial dilutions of mouse serum. Virus-serum mixtures were incubated for 1 h to allow virus-antibody binding. 100 μL of the virus-serum mixture was added to confluent 12-well plates in ascending dilution series and incubated for 1 h. Cells were overlaid with 0.5% agarose in growth medium and incubated for 72 h. After 72 h, cells were fixed in 4% formaldehyde, agarose plugs were removed, and cell monolayers were stained with 0.05% w/v crystal violet in 20% ethanol then washed in cold water. The PRNT titer was determined as the final dilution of the wells in which either an 80% or 50% reduction in plaques counted was observed versus virus diluent-virus control. Due to a limited serum quantity, PRNTs were not assayed in replicates.

### Laboratory vector competence

A colony of *Aedes aegypti* mosquitoes collected in Los Angeles, CA, was previously established^62^. Adults from generation F14 between 2- and 7-days old were divided into roughly equal cohorts by mechanical aspiration then sucrose-starved for 24 h prior to presentation to infectious bloodmeals. Male and female mosquitoes were exposed to heparinized sheep blood (Hemostat Laboratories) spiked with 10^5^–10^7^ PFU/mL of CHIKV LAV or WT at 1 part virus supernatant to two parts heparinized blood in a feeding apparatus (Hemotek Limited) warmed to 37 °C for a total of two 30-min sessions. Mosquitoes were cold-anesthetized and engorged females were sorted into disposable cardboard pint containers at a density of ≤10 per cage. Blood-fed mosquitoes were maintained at 26 °C and 80% relative humidity on a 12:12 h light-dark cycle with continuous access to 10% sucrose for the duration of the experiment. On day 10 post-feed, 30 mosquitoes per group were randomly selected and cold-anesthetized for salivation. Legs and wings were removed with forceps and the proboscis was guided into a sterile capillary tube dosed with ~2 µL of FBS to induce a salivation response. Capillary tubes were transferred after 15 min salivation time to microcentrifuge tubes with 400 µL of DMEM and centrifuged at >5000×*g* for 1 min to remove FBS from the capillary tube. Bodies were combined with dissected legs and wings and homogenized in 400 µL of DMEM with a glass bead at 26 beats/sec in a Tissuelyser (Retsch). Homogenates were centrifuged at >5000 × *g* for 4 min and supernatants and saliva samples were transferred to −80 °C to hold for virus titration by Vero plaque assay and qRT-PCR. A positive reading over the assay limit of detection was called a positive infection in the legs/wings/body or transmission in the saliva.

### Statistical analysis

Statistical testing was performed in GraphPad Prism except where noted for automated NGS sequencing analysis pipelines for ViVan and iVar^46,61^. Non-normal growth and PRNT data analyzed by two-way ANOVA were log_10_-transformed to pass normality assumptions. Dunnett’s test was selected for multiple comparisons with CHIKV-181/25 parental LAV as a control. Multiple comparisons of ANOVA were performed with Tukey’s test. Mouse mortality was assessed both by log-rank test and chi-square test for survival proportions. Similarly, mosquito infection and transmission rates were compared by chi-square test between cohorts.

### Reporting summary

Further information on experimental design is available in the Nature Research Reporting Summary linked to this paper.

## Supplementary information

Supplementary Files

Reporting Summary

## Data Availability

Sequences for newly described cDNA clones of CHIKV-181/25-P2.P4, CHIKV-181/25-P2.P4.IRES and CHIKV-181/25-P2.P4.E3/E1 (MT635335-MT635337) are available on Genbank. All raw deep sequencing data supporting Figs. 6 and 7 are available in fastq format on the NCBI Sequence Read Archive under BioProject PRJNA639833. All other data supporting the figures are available upon request from the corresponding author. Infectious clones first described here are available upon request from the corresponding author after completion of a Materials Transfer Agreement.
