# Supplementary material for: Engineering a fidelity-variant live-attenuated vaccine for chikungunya virus
Source: NPJ Vaccines. 2020 Oct 14;5:97. doi: 10.1038/s41541-020-00241-z (PMC7560698; doi:10.1038/s41541-020-00241-z)
Supplement: Supplementary file 1 — Supplementary Files [file 41541_2020_241_MOESM1_ESM.pdf]

nature research

Corresponding author(s): Lark L. Coffey

Last updated by author(s): Sep 1, 2020

Reporting Summary

Nature Research wishes to improve the reproducibility of the work that we publish. This form provides structure for consistency and transparency in reporting. For further information on Nature Research policies, see our Editorial Policies and the Editorial Policy Checklist.

Statistics

For all statistical analyses, confirm that the following items are present in the figure legend, table legend, main text, or Methods section.

n/a	Confirmed	
☐	☒	The exact sample size (*n*) for each experimental group/condition, given as a discrete number and unit of measurement
☐	☒	A statement on whether measurements were taken from distinct samples or whether the same sample was measured repeatedly
☐	☒	The statistical test(s) used AND whether they are one- or two-sided<br>*Only common tests should be described solely by name; describe more complex techniques in the Methods section.*
☐	☒	A description of all covariates tested
☐	☒	A description of any assumptions or corrections, such as tests of normality and adjustment for multiple comparisons
☐	☒	A full description of the statistical parameters including central tendency (e.g. means) or other basic estimates (e.g. regression coefficient) AND variation (e.g. standard deviation) or associated estimates of uncertainty (e.g. confidence intervals)
☐	☒	For null hypothesis testing, the test statistic (e.g. *F*, *t*, *r*) with confidence intervals, effect sizes, degrees of freedom and *P* value noted<br>*Give P values as exact values whenever suitable.*
☒	☐	For Bayesian analysis, information on the choice of priors and Markov chain Monte Carlo settings
☒	☐	For hierarchical and complex designs, identification of the appropriate level for tests and full reporting of outcomes
☒	☐	Estimates of effect sizes (e.g. Cohen's *d*, Pearson's *r*), indicating how they were calculated

Our web collection on statistics for biologists contains articles on many of the points above.

Software and code

Policy information about availability of computer code

Data collection: *Provide a description of all commercial, open source and custom code used to collect the data in this study, specifying the version used OR state that no software was used.*

Data analysis: *Provide a description of all commercial, open source and custom code used to analyse the data in this study, specifying the version used OR state that no software was used.*

For manuscripts utilizing custom algorithms or software that are central to the research but not yet described in published literature, software must be made available to editors and reviewers. We strongly encourage code deposition in a community repository (e.g. GitHub). See the Nature Research guidelines for submitting code & software for further information.

Data

Policy information about availability of data

All manuscripts must include a data availability statement. This statement should provide the following information, where applicable:

- Accession codes, unique identifiers, or web links for publicly available datasets
- A list of figures that have associated raw data
- A description of any restrictions on data availability

Sequences for cDNA clones of CHIKV-181/25-P2.P4, CHIKV-181/25-P2.P4.IRES and CHIKV-181/25-P2.P4.E3/E1 (MT635335-MT635337) are available on Genbank. All raw deep sequencing data is available in fastq format on the NCBI Sequence Read Archive under BioProject PRJNA639833.

Field-specific reporting

Please select the one below that is the best fit for your research. If you are not sure, read the appropriate sections before making your selection.

☒ Life sciences ☐ Behavioural & social sciences ☐ Ecological, evolutionary & environmental sciences

For a reference copy of the document with all sections, see nature.com/documents/nr-reporting-summary-flat.pdf

Life sciences study design

All studies must disclose on these points even when the disclosure is negative.

Sample size	Sample sizes calculations were not performed. Sample sizes were chosen to balance male and female animal subjects and mirror previous studies of similar design.
Data exclusions	No data were excluded from analysis.
Replication	Experiments were replicated where possible and data is shown from two replicate experiments. Virus passage experiments were completed one time.
Randomization	Animals were randomized from combined litters and redistributed to a nursing dam at birth. Older mice were sorted arbitrarily with the same sex and acclimated with selected cage mates prior to experiment initiation.
Blinding	Investigators were not blinded to the identity of experimental groups due to cage labeling requirements for the pathogens present in the study. For experiments where longitudinal data was collected (i.e. measuring footpad area with calipers), measurements were collected and recorded by different investigators to avoid biasing measurements to fit a trend.

Reporting for specific materials, systems and methods

We require information from authors about some types of materials, experimental systems and methods used in many studies. Here, indicate whether each material, system or method listed is relevant to your study. If you are not sure if a list item applies to your research, read the appropriate section before selecting a response.

Materials & experimental systems

n/a	Involved in the study	
☒	☐	Antibodies
☐	☒	Eukaryotic cell lines
☒	☐	Palaeontology and archaeology
☐	☒	Animals and other organisms
☒	☐	Human research participants
☒	☐	Clinical data
☒	☐	Dual use research of concern

Methods

n/a	Involved in the study	
☒	☐	ChIP-seq
☒	☐	Flow cytometry
☒	☐	MRI-based neuroimaging

Eukaryotic cell lines

Policy information about cell lines

Cell line source(s)	Purchased from ATCC and minimally passaged prior to use
Authentication	Cell lines were not authenticated
Mycoplasma contamination	Cells were assessed for mycoplasma contamination on a quarterly schedule using PCR-based detection assay.
Commonly misidentified lines (See ICLAC register)	*Name any commonly misidentified cell lines used in the study and provide a rationale for their use.*

Animals and other organisms

Policy information about studies involving animals; ARRIVE guidelines recommended for reporting animal research

Laboratory animals	House mouse (mus musculus), CD1-IGS neonates (not sexed) aged 2-6 days old, C57Bl/6J male and female (equal ratio) 6-weeks of age
Wild animals	*Provide details on animals observed in or captured in the field; report species, sex and age where possible. Describe how animals were*

Wild animals	*caught and transported and what happened to captive animals after the study (if killed, explain why and describe method; if released, say where and when) OR state that the study did not involve wild animals.*
Field-collected samples	*For laboratory work with field-collected samples, describe all relevant parameters such as housing, maintenance, temperature, photoperiod and end-of-experiment protocol OR state that the study did not involve samples collected from the field.*
Ethics oversight	University of California, Davis, Institutional Animal Care and Use Committee -- Protocol 20966.

Note that full information on the approval of the study protocol must also be provided in the manuscript.
