# Supplementary material for: Engineering a fidelity-variant live-attenuated vaccine for chikungunya virus
Source: NPJ Vaccines. 2020 Oct 14;5:97. doi: 10.1038/s41541-020-00241-z (PMC7560698; doi:10.1038/s41541-020-00241-z)
Supplement: Supplementary file 2 — Reporting Summary [file 41541_2020_241_MOESM2_ESM.pdf]

SUPPLEMENTARY FIGURES

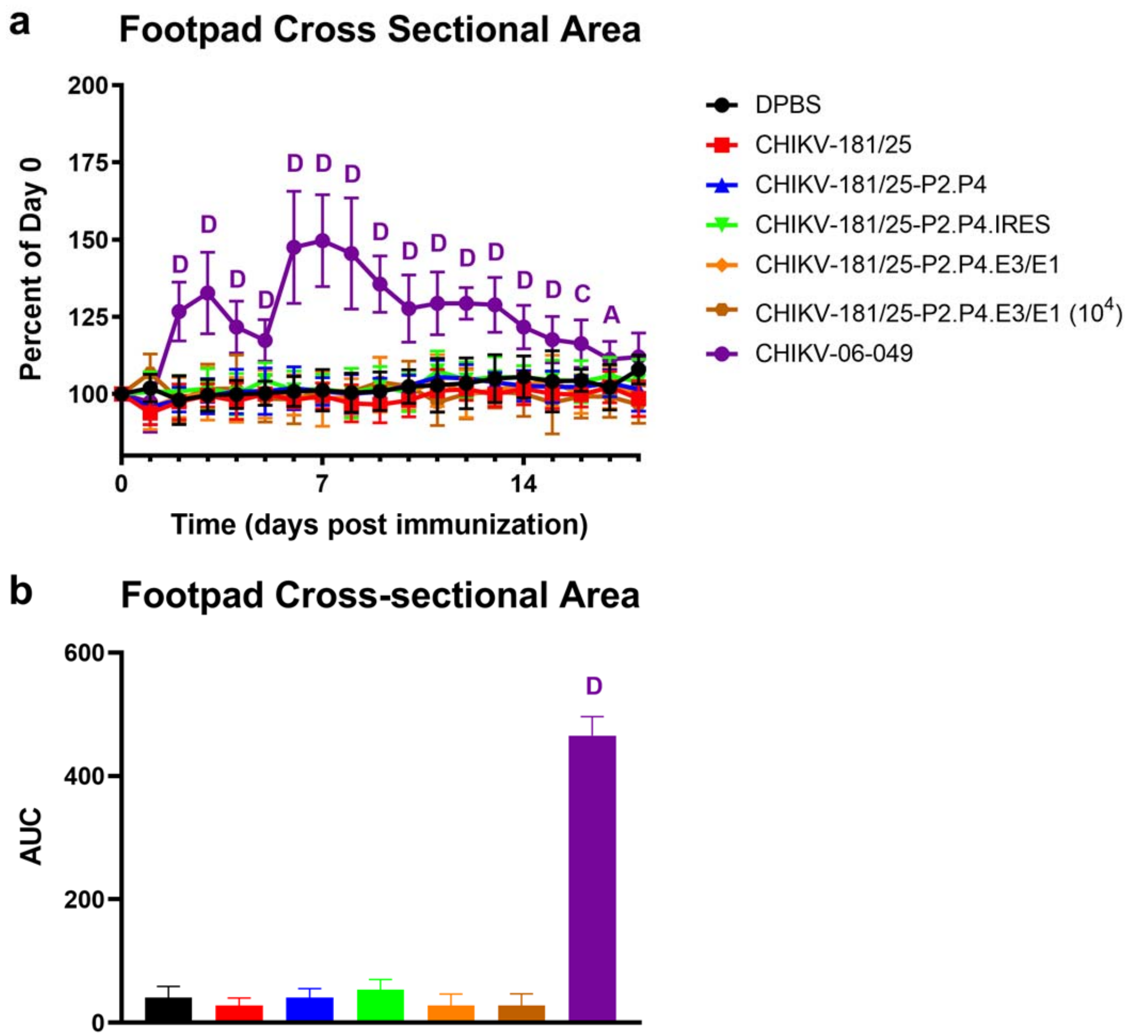

Supplementary Figure 1: Ipsilateral footpad swelling associated with LAV CHIKV immunization. Adult C57Bl/6J mice were immunized with 10^3 PFU CHIKV LAV derivatives unless indicated as 10^4 PFU. Hindlimb footpad cross-sectional area was measured as an ellipse once daily for 18 days post inoculation (**a**) and total swelling area under the curve (**b**) are shown post immunization. *P* < 0.05 (A), 0.001 (C), 0.0001 (D), 2-Way ANOVA on untransformed values, n=5 per experiment, 2 combined experiments. Mean and standard deviation are indicated with data points/bars and error bars. AUC=Area Under the Curve.

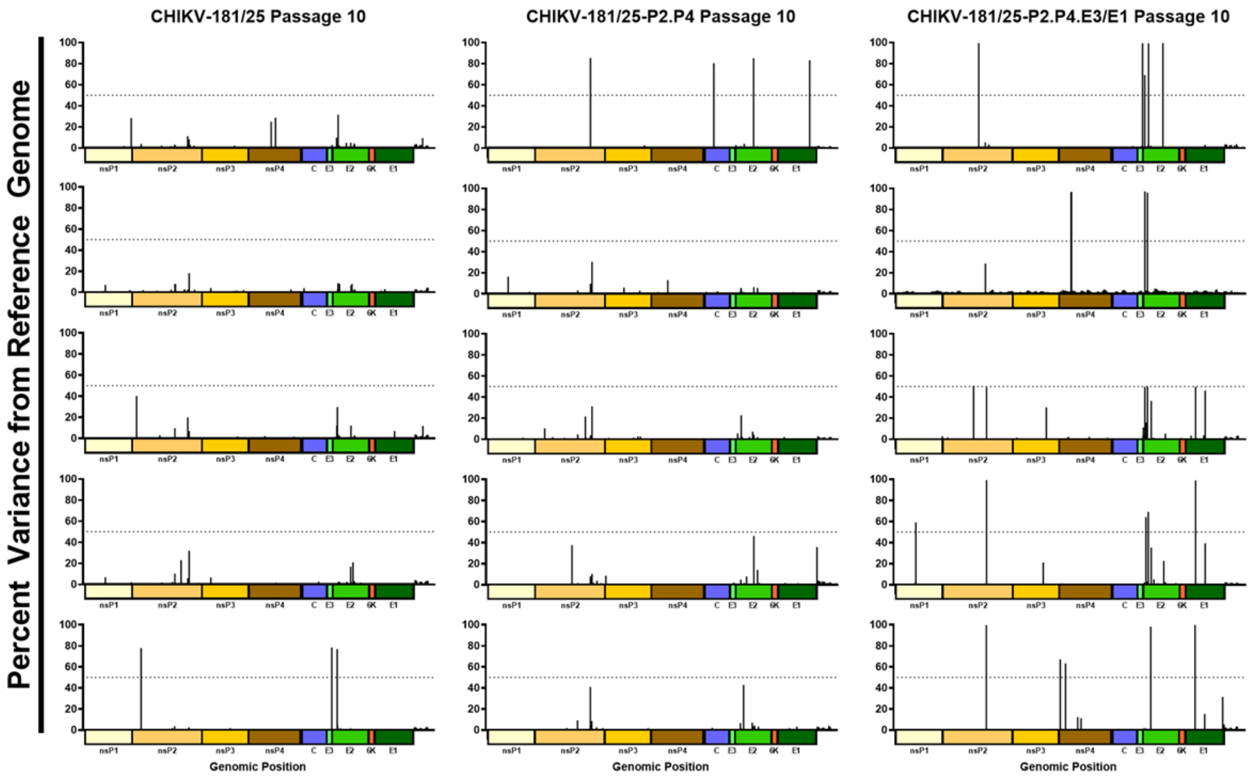

Supplementary Figure 2: Mutation accumulation of cell culture-passaged fidelity-variant CHIKV live-attenuated vaccine candidates. Positions in genome and frequency of single nucleotide polymorphisms acquired over 10 serial passages in BHK-21 cells for each CHIKV live-attenuated vaccine are given. Each graph represents a one replicate passage. Dashed line is 50% of fragments sequenced, indicating a consensus change for the population. Data are percent variance from the reference genome for individual replicates of serially passaged CHIKV LAV.

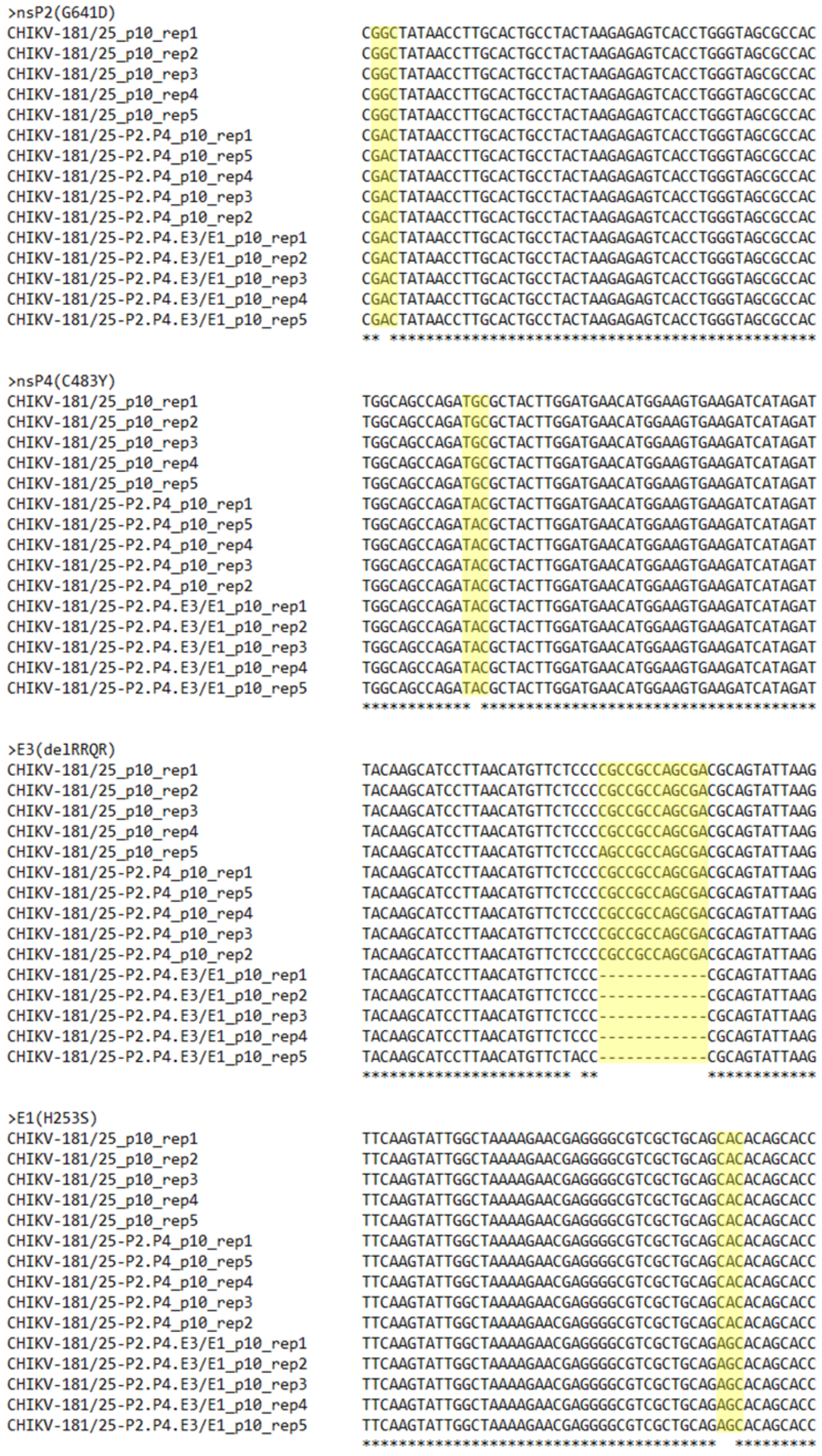

Supplementary Figure 3: Consensus sequence of engineered mutation sites following serial cell culture passage. Consensus sequences of CHIKV LAVs following serial cell culture passage in BHK-21 cells were aligned with Clustal W algorithm. Engineered nucleotide mutations are highlighted in yellow. Asterisks indicate consensus multiple alignment, gaps indicate mixed multiple alignment, and dashes indicate deleted nucleotides.

SUPPLEMENTARY TABLES

Primer Name	Pool #	Primer Sequence
CV181-25_1_LEFT	1	GAGACACACGTAGCCTACCAGT
CV181-25_1_RIGHT	1	CGCTTGTAAGTCCCCGATCTTT
CV181-25_2_LEFT**	2	TGTCGGACAGGAAGTACCACTG
CV181-25_2_RIGHT**	2	TTGTCGAGTATGAGGGGTAGGC
CV181-25_3_LEFT	1	ACCACCAGGCGATTAAAGGAGT
CV181-25_3_RIGHT	1	CACACGAAACCACTGTGTCACA
CV181-25_4_LEFT	2	TGTTCTCAGTAGGGTCAACGCT
CV181-25_4_RIGHT	2	ACCACTATCCTCTGGTTCAGCC
CV181-25_5_LEFT	1	CCGGCGACCATTTGTGATCAAA
CV181-25_5_RIGHT	1	GTCCTCAACGGGATTGACAACC
CV181-25_6_LEFT	2	ACAAGAGGCCTGATACCCAGTC
CV181-25_6_RIGHT	2	CACGACGTGGTCTGTTAGTTGG
CV181-25_7_LEFT	1	CGAAATCGACGTGGAACAGCTT
CV181-25_7_RIGHT	1	CCTCACCAGCTCATACGACTCT
CV181-25_8_LEFT	2	AAAGCGCAACGATGGTGTACAA
CV181-25_8_RIGHT	2	CTCTCTGTCTCATCACGTCGGT
CV181-25_9_LEFT	1	GCAAGTCAGCCATTATCAAGAACC
CV181-25_9_RIGHT	1	CAGTGTACACCGCCTGGAGATA
CV181-25_10_LEFT	2	AGCAGTGCGGCTTCTTCAATAT
CV181-25_10_RIGHT	2	GACGTGCTCTGATGTTGATGCA
CV181-25_11_LEFT	1	ACTGCAAATTGACTATCGTGGACA
CV181-25_11_RIGHT	1	TTATCCCCGCTGTTTCGAGGAT
CV181-25_12_LEFT	2	CATCGATAATGGCGGGCATCTG
CV181-25_12_RIGHT	2	TCCTGGTAGTCACGCAGATCTG
CV181-25_13_LEFT	1	TTCGGATTCAACCCTGAGGCAG
CV181-25_13_RIGHT	1	GGTCATACCTACCAAGCGTTGC
CV181-25_14_LEFT	2	TAAGAGAGTCACCTGGGTAGCG
CV181-25_14_RIGHT	2	AGGCTGCATTCAGTTGATTGTTCA
CV181-25_15_LEFT	1	GAAACCACCATGTGTCACCAGT
CV181-25_15_RIGHT	1	CCTTTGCGACTTCTCGATAGGC
CV181-25_16_LEFT	2	CGTATCCAGTAATCCACGCCGT
CV181-25_16_RIGHT	2	TTTTCTGCCTGCCAAGCTACTG
CV181-25_17_LEFT	1	CATACAGATGCGGACCCAAGTG
CV181-25_17_RIGHT	1	TGACATGGTTCATGCGAAGTCG
CV181-25_18_LEFT	2	CAGAAATGCCCGGTGGATGATG
CV181-25_18_RIGHT	2	GTAATGTATGTACCGCCCCGTC
CV181-25_19_LEFT	1	AGTTTGATCTAAGCGCCGATGG

CV181-25_19_RIGHT	1	CCTTCGTCGAAGTCCCCAAATG
CV181-25_20_LEFT	2	CAGCTATTTCCCTTCAGGCACC
CV181-25_20_RIGHT	2	GGGTAACACTTCTCCTCGTGGA
CV181-25_21_LEFT	1	GACACTGGTCCAGGCCATTTAC
CV181-25_21_RIGHT	1	ACTCATTACATGCTGCCACTGC
CV181-25_22_LEFT	2	CGACTTATCGGACCATATACCCG
CV181-25_22_RIGHT	2	ACTGCTGAGTCCAAAGTGGGTA
CV181-25_23_LEFT	1	ACCTTCCCCATTCCAGAACACA
CV181-25_23_RIGHT	1	CATAGGTACGCTGTTGCCAAGG
CV181-25_24_LEFT	2	CAGTAGATATGAAAAGGGATGTGAAGGT
CV181-25_24_RIGHT	2	AATCTCTCCGAAAGCAGCCTCT
CV181-25_25_LEFT	1	GCCAAGATGATTCACTTGCGCT
CV181-25_25_RIGHT	1	GGAGCTTTCTGGGATACAACTGC
CV181-25_26_LEFT	2	ACATGGTGTCGTCTCCGATGAA
CV181-25_26_RIGHT	2	GCAAAGGTGGCCATGGACATTA
CV181-25_27_LEFT	1	GATGGCAACGAACAGGGCTAAT
CV181-25_27_RIGHT	1	CTGAGATCAGCTGGGCAAGTTG
CV181-25_28_LEFT	2	TTCTACAATAGGAGGTACCAGCCT
CV181-25_28_RIGHT	2	ATCGATGGTCCCCTTTACGTGT
CV181-25_29_LEFT	1	TGCATCTTCGAAGTCAAGCATGA
CV181-25_29_RIGHT	1	TACGGGCTCCTTCATTAGCTCC
CV181-25_30_LEFT*	2	TACAGTACTCAGGAGGCCGGTT
CV181-25_30_RIGHT_V2*	2	TGGCTTTATAGACATTGAAGTTGTCC
CV181-25_31_LEFT	1	CGCATGCTAGAAGACAACGTCA
CV181-25_31_RIGHT	1	GCCAGGATGAAGTGTCCCATTG
CV181-25_32_LEFT	2	AATCATATGCCAGCAGACGCAG
CV181-25_32_RIGHT	2	TGTACCGCACCGTCTGACTATT
CV181-25_33_LEFT	1	AGGTACATATGCCCCCAGACAC
CV181-25_33_RIGHT	1	TGGTCAGGATACAGCAGCATGA
CV181-25_34_LEFT	2	TCCTCTGGCAAATGTGACATGC
CV181-25_34_RIGHT	2	ATCGACAGGAGTACGAACGAGG
CV181-25_35_LEFT	1	GGCCACCCGCATGAGATAATTT
CV181-25_35_RIGHT	1	CCGACGCTCATTACGGCTAAAA
CV181-25_36_LEFT	2	AACGAGCAGCAGCCTTTGTTTT
CV181-25_36_RIGHT	2	CTCTTGTCCTTGCACTCTGCTG
CV181-25_37_LEFT	1	AGCCAACGCTATCGCTTGATTA
CV181-25_37_RIGHT	1	GTGTCCAGGCTGAAGACATTGG
CV181-25_38_LEFT	2	ACCAAGGAAATAATGTTACTGTATCTGCT
CV181-25_38_RIGHT	2	AGTTCATCGCTCTTACCGGGTT
CV181-25_39_LEFT	1	GGCACCATCTGGCTTCAAGTAT
CV181-25_39_RIGHT	1	ACAGACTTGTACGCGGAATTCG

CV181-25_40_LEFT	2	TAACGCCGTCACTATTCGGGAA
CV181-25_40_RIGHT_V2	2	GGGACACATATACCTTCGTACC
CV181-25_41_LEFT	1	GCAGCACTGATCCTAATCGTGG
CV181-25_41_RIGHT	1	AGCCCTTTGAACTACTTCTGTTTATGG
CV181-25_42_LEFT_V2**	2	GAGACACACCATATATAGCTAAGAATCAAT
CV181-25_42_RIGHT**	2	ACCTACATCTCTTCCGTTTGCC

Supplementary Table 1. Primers used for PrimalSeq amplicon-based sequencing of CHIKV-181/25 genomes. Primers were generated using PrimalScheme wrapper for Primer3 multiplex primer design tool targeting the CHIKV-181/25 full-length genome [47]. Variant (V2) primers were modified manually to account for gaps in CHIKV-181/25-P2.P4.E3/E1 and regions of high similarity in conserved repeat elements present in the CHIKV 3'-UTR. All primers were pooled with equal volumes of 10 µM starting concentration unless noted otherwise (* 50 µM, ** 100 µM).

Score	Short Description	Long Description
**0**	none	no lesions observed
**1**	minimal	focal or scattered, minimal, subcutaneous inflammation and/or edema not observed in control animals or of questionable significance; could be a background lesion or variation of normal
**2**	mild	mild inflammation of subcutaneous tissue, fascia, and/or skeletal muscle affecting less than 10% of the soft tissue surrounding tarsus/metatarsus/digit, and/or moderate to severe edema of subcutaneous tissue
**3**	moderate	mild to moderate inflammation and edema of subcutaneous tissue, fascia, skeletal muscle, and/or tendon affecting 10-25% of the soft tissue surrounding tarsus/metatarsus/ digit; and/or scattered myocyte necrosis; and/or synovial cell reactive hyperplasia
**4**	severe	widespread, severe inflammation and edema of subcutaneous tissue, fascia, skeletal muscle, and/or tendon affecting greater than or equal to 25% of the soft tissue surrounding tarsus/metatarsus/digit, and/or more widespread myocyte necrosis; and/or synovial cell reactive hyperplasia or mild synovial/peri-synovial inflammation
**5**	severe with evidence of joint lesions	same as “4” but with more severe inflammation; and/or inflammation/necrotic debris in the joint space; and/or lesions affecting articular cartilage or bone

Supplementary Table 2: Pathology scoring criteria.
